# Low interannual precipitation has a greater negative effect than seedling herbivory on the population dynamics of a short‐lived shrub, *Schiedea obovata*

**DOI:** 10.1002/ece3.3595

**Published:** 2017-11-26

**Authors:** Lalasia Bialic‐Murphy, Orou G. Gaoue

**Affiliations:** ^1^ Department of Ecology and Evolutionary Biology University of Tennessee Knoxville Knoxville TN USA; ^2^ Department of Botany University of Hawai'i at Manoa Honolulu HI USA; ^3^ Faculty of Agronomy University of Parakou Parakou Benin; ^4^ Department of Geography, Environmental Management and Energy Studies University of Johannesburg Johannesburg South Africa

**Keywords:** endangered species, integral projection model, interannual precipitation, life table response experiment, mollusk herbivory, plant reintroduction, plant–animal interactions, plant–climate interactions, restoration ecology, temporal variability

## Abstract

Climate projections forecast more extreme interannual climate variability over time, with an increase in the severity and duration of extreme drought and rainfall events. Based on bioclimatic envelope models, it is projected that changing precipitation patterns will drastically alter the spatial distributions and density of plants and be a primary driver of biodiversity loss. However, many other underlying mechanisms can impact plant vital rates (i.e., survival, growth, and reproduction) and population dynamics. In this study, we developed a size‐dependent integral projection model (IPM) to evaluate how interannual precipitation and mollusk herbivory influence the dynamics of a Hawaii endemic short‐lived shrub, *Schiedea obovata* (Caryophyllaceae). Assessing how wet season precipitation effects population dynamics it critical, as it is the timeframe when most of the foliar growth occurs, plants flower and fruit, and seedlings establish. Temporal variation in wet season precipitation had a greater effect than mollusk herbivory on *S*. *obovata* population growth rate λ, and the impact of interannual precipitation on vital rates shifted across plant ontogeny. Furthermore, wet season precipitation influenced multiple vital rates in contrasting ways and the effect of precipitation on the survival of larger vegetative and reproductively mature individuals contributed the most to variation in the population growth rate. Among all combination of wet season precipitation and herbivory intensities, the only scenario that led to a growing population was when high wet precipitation was associated with low herbivory. Our study highlights the importance of evaluating how abiotic factors and plant–consumer interactions influence an organism across its life cycle to fully understand the underpinning mechanisms that structure its spatial and temporal distribution and abundance. Our results also illustrate that for short‐lived species, like *S. obovata*, seedling herbivory can have less of an effect on the dynamics of plant populations than decreased interannual precipitation.

## 1 INTRODUCTION

Plant endangerment is driven by complex interactions of multiple environmental stressors, which can have varying effects on plant vital rates and ultimately population dynamics (Brook, Sodhi, & Bradshaw, 2008). Two environmental stressors implicated in the decline of rare species are changing precipitation patterns and the introduction of non‐native herbivores (Parmesan, 2006; Wilcove, Rothstein, Dubow, Phillips, & Losos, 1998). The independent influences of these environmental stressors on various components of plant fitness (e.g., seedling survival) have been well examined and often used to prioritize restoration actions (Cowie, Dillon, Robinson, & Smith, 2009; Hanley, Fenner, & Edwards, 1995; Joe & Daehler, 2007; Orians, Fritz, Hochwender, Albrectsen, & Czesak, 2013; Parmesan, 2006; Pender, Shiels, Bialic‐Murphy, & Mosher, 2013). From those studies, we have gained an in‐depth understanding of the direct impacts of various environmental stressors on targeted vital rates of native species. However, comparing the effects of different environmental stressors on part of a species life cycle can be a poor predictor of which environmental stressors will have the greatest effect on population growth rate and can lead to misleading management recommendations (Ehrlén, 2003).

A primary environmental factor implicated in the decline of species globally is changing abiotic conditions, including more severe drought and rainfall events (Parmesan, 2006). Climate projection models suggest that large‐scale environmental change has already directly affected the phenology and geographical range of Earth's flora and fauna (Guisan & Thuiller, 2005). It is predicted that environmental conditions will continue to drastically alter species' distributions, as variation in interannual environmental conditions increases (Guisan & Thuiller, 2005). A primary assumption of those studies and future projections of the influence of environmental change on species' climate envelope (i.e., projected distribution models) is that abiotic parameters, including precipitation, are strong drivers of the spatial distribution and abundance of plants (Guisan & Thuiller, 2005). However, climate change can have varying effects on plant vital rates across ontogeny, sometimes in opposite directions. Other underlying mechanisms, such as plant–consumer interactions, can also influence the spatial distribution and abundance of plants along an elevation gradient (Miller, Louda, Rose, & Eckberg, 2009). Surprisingly, few studies have investigated how changes in precipitation influence the full life cycle of plants and quantified how precipitation drives spatial and temporal variation in population dynamics (but see, Dalgleish, Koons, & Adler, 2010; Dalgleish, Koons, Hooten, Moffet, & Adler, 2011; Jongejans, De Kroon, Tuljapurkar, & Shea, 2010; Salguero‐Gómez, Siewert, Casper, & Tielbörger, 2012; Tye, Menges, Weekley, Quintana‐Ascencio, & Salguero‐Gómez, 2016; Williams, Jacquemyn, Ochocki, Brys, & Miller, 2015).

In addition to changes in climate, selection pressures from non‐native herbivores are critical drivers of species endangerment and exogenous determinants of plant fitness and evolutionary potential. Due to the lack of diverse herbivore communities on remote islands, many island endemic species have lower mechanistic and physiologic tolerance to non‐native herbivores than continental relatives (Bowen & Vuren, 1997; Carlquist, 1974; Vitousek, 1988; Vourc'h, Martin, Duncan, Escarré, & Clausen, 2001). Thus, non‐native herbivores may have a disproportionally large effect on the spatial distribution and abundance of island species. Selection pressure by herbivores can lead to increased plant tolerance and resilience in subsequent generations (Orians et al., 2013). At the community level, herbivores can decrease plant diversity (Hanley et al., 1995). Most herbivore–plant interaction studies, however, have not explicitly evaluated how changes in various measures of plant vital rates (e.g., survival, growth, and reproduction) influence the overall population dynamics (but see, Ehrlen, 1995a, 1995b; Maron & Crone, 2006; Miller et al., 2009). Of the studies that have been conducted to evaluate the population level effects of plant–herbivore interactions most have focused on vertebrates and insects, which primarily reduce vegetative biomass of later life stages (e.g., adults) and fertility, respectively (Maron & Crone, 2006). There is also a small, but growing, body of literature that highlights how plant–herbivore interactions can vary along an geographical gradient, suggesting plant–herbivore interactions are context specific (Dahlgren & Ehrlén, 2009; Miller et al., 2009).

Some of the most ubiquitous herbivores implicated in the decline of species, particularly on tropical oceanic islands, are non‐native mollusks (Cowie et al., 2009; Joe & Daehler, 2007; Lowe, Browne, Boudjelas, & De Poorter, 2000). Mollusks primarily influence plant vital rates of earlier life stages (i.e., seedling establishment and survival), consuming meristematic tissue and foliage at the ground level (Barker, 1989; Byers & Bierlein, 1982). Following mollusk suppression, seedling establishment and density can increase significantly (Ehrlén, 2003; Hanley et al., 1995; Joe & Daehler, 2007). Interestingly, mollusk herbivory can have a larger effect on population growth rate than vertebrate grazers and pre‐ and postseed predators (Ehrlen, 1995a, 1995b; Ehrlén, 2003). However, there is a paucity of studies that have explicitly evaluated how mollusk herbivory influences the dynamics of plant populations, and this limits our ability to make guild‐specific comparisons. A pressing question in conservation is whether changing abiotic conditions or introduced herbivores are having a greater negative effect on the dynamics of endangered species. This is particularly true for oceanic island ecosystems, which are biodiversity hot spots and harbor some of the highest rates of species endangerment and extinction.

In this study, we constructed a precipitation‐ and herbivory‐dependent integral projection model (IPM) to investigate how wet season precipitation and seedling herbivory by non‐native mollusks (*Stylommatophora*, Limacidae and *Systellommatophora*, Veronicellidae) affect the temporal variation in the dynamics of a reintroduced population of a short‐lived Hawaii endemic shrub, *Schiedea obovata* (Caryophyllaceae). Total annual precipitation can mask the effect of seasonal interannual precipitation on plant vital rates (i.e., survival, growth, and fertility). For this reason, we focused on evaluating the effect of temporal variation in wet season precipitation (October–March), which is when the focal taxon, *S. obovata*, is fruiting, seedlings are establishing, and most vegetative growth occurs. To assess how wet season precipitation and herbivory influence the population dynamics of *S. obovata,* we asked the following questions: (1) Does interannual precipitation or herbivory have a greater effect on *S. obovata* vital rates and population dynamics? (2) What demographic processes (i.e., survival, growth, and fertility) drive differences in population growth rates between years that vary in wet season precipitation?

## 2 MATERIAL AND METHODS

### 2.1 Study species

*Schiedea obovata* is a suberect or ascending Hawaii endemic short‐lived, perennial, shrub, reaching 0.3–1 m tall (Wagner, Weller, & Sakai, 2005). Fruits are capsules, and the sepals are fleshy and dark purple. The pollen:ovule ratio of 310 for *S. obovata* indicates its breeding system is somewhere between facultative autogamy and xenogamy (Wagner et al., 2005). The purple berries it produces are indicative of frugivorous bird dispersal. *Schiedea obovata* fruits and flowers at the end of wet season from February to May and seedlings establish the following wet season from December to March. The historical range of *S. obovata* spanned the Waianae Mountain Range, on the island of O'ahu, Hawai'i (Wagner et al. 1999). Over the past several decades, *S. obovata* has experienced a severe reduction in geographical range, and by 1991, it was listed as federally endangered (USFWS 1991).

### 2.2 Study site and reintroduction details

The study site was a reintroduced population of *S. obovata* that is located in the Kahanahaiki Management Unit (36 ha), referred to hereafter as Kahanahaiki, which is in the northern Waianae Mountain Range, on the island of O'ahu (21°32′N, −158°12′W). Kahanahaiki is a tropical mesic forest; composed of a mix of native and non‐native flora and fauna. In 1996, prior to reintroduction, the O'ahu Army Natural Resources Program (OANRP) constructed the Kahanahaiki fence and controlled non‐native ungulates. From 1999 to 2011, a total of six *S. obovata* outplanting efforts were undertaken and 258 individuals were reintroduced. Since 1998, OANRP has been conducting ecosystem‐level management for the protection of *S. obovata* and eleven other endangered species (Garrison, US Army 2007), which includes the control of a non‐native rodent *Rattus rattus* and bi‐annual weed control of competitive ecosystem altering vegetation. Starting in 2011, localized suppression of non‐native mollusks (*Stylommatophora*, Limacidae and *Systellommatophora*, Veronicellidae) has been executed at the reintroduction site. A monthly application rate of a molluscicide, Sluggo (Neudorff Co., Fresno, California), was used as the suppression methodology. Sluggo was not applied in the summer months, when mollusk herbivory was minimal (S. Joe, personal communication).

The stock used to establish the Kahanahaiki *S. obovata* population came from a single founder growing in close proximity to the reintroduction site. Seeds used for the reintroduction were collected and grown in a greenhouse for one growing season, prior to outplanting. The mean height of *S. obovata* when outplanted at Kahanahaiki was 58 cm. Site selection of the reintroduction was based on the following criteria: (1) appropriate habitat and associated species, (2) similar topography as naturally occurring populations and, (3) geographical proximity to naturally occurring *S. obovata* individuals (Garrison, US Army 2007). Genetic stock from the other six known populations was not used for the Kahanahaiki reintroduction to avoid potential outbreeding depression and the loss of localized adaptations (Kawelo, Harbin, Joe, Keir, & Weisenberger, 2012). That decision was partially supported by recent research that examined the risk of inbreeding and outbreeding depression of mixed founder stock (Weisenberger, 2012). Inbreeding and outbreeding depression were not detected. However, plants from maternal source populations furthest from Kahanahaiki had the lowest progeny fitness when outplanted at Kahanahaiki. Given that Kahanahaiki was the driest and lowest elevation site that *S. obovata* had been documented from, reduced progeny fitness of plants from maternal source populations furthest from Kahanahaiki may indicate local adaptation. Alternatively, reduced progeny fitness may be the effect of the small population size and genetic drift. The management recommendation of Weisenberger (2012) for the reintroduction of *S. obovata* at Kahanahaiki was: “propagules that originate from higher elevations should not be moved to lower elevations. Kahanahaiki progeny are the only plants that should be planted into Kahanahaiki gulch.”

### 2.3 Demographic data

Demographic field monitoring was initiated in April of 2014 and was conducted annually for three consecutive years. Over the study period, we installed 18 1 × 1 m permanent plots throughout the reintroduction field site and collected demographic data for a total of 422 individual plants. Plants in each plot that were >8 cm in height were permanently tagged. For plants <8 cm, a subset of ten randomly selected plants was permanently tagged in each plot. To avoid damage, plants <8 cm were marked using pin flags and color coded wire. To evaluate the potential effect of density dependence on seedling vital rates, we assessed differences in the survival and growth of seedling clusters and isolated seedlings. For each tagged plant, we recorded its height to apical meristem, basal diameter, survival, and reproductive status (the presence of flowers and fruits).

### 2.4 Construction of precipitation‐dependent vital rate functions

To explicitly evaluate the effects of wet season precipitation on the survival, growth, and fertility functions (see Equations (2) and (3)), we used a generalized linear mixed‐effect model with precipitation and plant size at time *t* as predictor variables and plot and plant ID as random effects (Bolker et al., 2009). To investigate all potential effects and interactions of wet season precipitation and the associated vital rate predictor variables we used model selection, starting with a fully parameterized model and Akaike Information Criterion (AIC) to select the best‐supported models. To include all potential effects and interactions, we compared ΔAICc values and selected the most complex model with a ΔAICc < 2, where ΔAICc is the difference in AIC corrected for sample size between each candidate model and the model with the lowest AICc value. For the survival *s* $\left(x,\;a,\;b\right)$ model and the probability of fruiting function $f_{f}\left(x,\;a,\;b\right)$ of the fertility model we used a binomial error structure and for the growth model g $\left(y,\;x,\;a,\;b\right)$ we used a normal error structure. For the reproductive output function $p_{r}\left(a,\;b\right)$ (i.e., number of seedling per mature plant) of the fertility model, we used a negative binomial error structure to account for overdispersion.

The precipitation data (i.e., parameter *a*) that we used to evaluate the effect of temporal variation in wet season precipitation on plant vital rates and population dynamics were collated from the National Oceanic and Atmospheric Administration for the Honolulu airport meteorological station 21.324°N and 157.929°W (NOAA Regional Climate Center, 2016). The first wet season 2014–2015 had average precipitation (10.4 in), and the second wet season 2015–2016 had lower than average precipitation (5.99 in) and was ranked a very strong (SST > 2) El Nino Southern Oscillation Year (NOAA Regional Climate Center, 2016). For simplicity, hereafter the first wet season 2014–2015 is referred to as *high wet season precipitation (HP)* and the second wet season 2015–2016 is referred to as *low wet season precipitation (LP)*.

The best‐supported generalized linear mixed‐effect models for survival *s*(*x*,* a*) and probability of fruiting $f_{f}\left(x,\;a\right)$ were the most complex models, including initial size, inter‐annual precipitation, and their interaction as predictor variables (Table 1). The best‐supported model for plant growth $g\left(x,\;a,\;b\right)$ included the initial plant size as the predictor variable, suggesting that precipitation has little or no effect on growth within the bounds of precipitation that occurred over the study period 2014–2016 (Table 1). For the reproductive output function $p_{r}\left(a,\;b\right)$, the best‐supported model was using wet season precipitation as the predictor variable (Table 1).

**Table 1:** Generalized linear mixed‐effect models of survival *s* (*x, a, b*), growth *g* (*x, a, b*), probability of fruiting $f_{f}\left(x,\;a,\;b\right)$, and reproductive output $p_{r}\left(a,\;b\right)$. The models in gray represent the most complex model with a ΔAICc < 2. Size = height to apical meristem, number of seedlings = number of seedling at time *t* per mature plant at *t* + 1, and precipitation represents total wet season precipitation. For all models, plot and plant ID were included as random effects

Estimate *t* ΔAICc							
		Intercept	Size	Precipitation	Size × Precipitation	*df*	ΔAICc
Survival	Size	−1.85991	0.92151	—	—	4	6.9
	Size + precipitation	−2.44426	0.94404	0.06794	—	5	7.4
	Size × precipitation	−0.76798	0.05506	−0.15571	0.11733	6	0.0
Growth	Size	1.49416	0.65157	—	—	5	0.0
	Size + precipitation	1.66553	0.64858	−0.02049	—	6	6.5
	Size × precipitation	1.95211	0.53731	−0.05994	0.01509	7	13.9
Probability of flowering	Size	−10.2862	2.7757	—	—	4	7.7
	Size + precipitation	−13.90104	3.15171	0.28373	—	5	0.0
	Size × precipitation	−9.2727	1.8207	−0.3061	0.1723	6	1.0
Number of seedlings	Size	−1.4738	0.4136	—	—	5	14.8
	Precipitation	−0.4156	—	0.07283		5	0.0
	Size + precipitation	−4.67704	0.86991	0.15256	—	6	11.2
	Size × precipitation	4.7743	−1.6355	−0.9349	0.2882	7	4.7

### 2.5 Effect of mollusk herbivory on *S. obovata* vital rates

To evaluate the effects of mollusk herbivory on the survival, growth, and fertility of *S. obovata,* we used a combination of field experiments. The effect of mollusk herbivory on seedling survival came from a field experiment that was conducted in close proximity to our study site (Kawelo et al., 2012). In that study, *S. obovata* seeds were sown on the top layer of soil in 12 plots, 15 m × 15 m in diameter. Six of the plots were treated with molluscicide, Sluggo (Neudorff Co., Fresno, CA, USA), and the other six plots were left exposed to field herbivory conditions. Seedling density was recorded on a weekly basis for 6 weeks. The results of that study indicated that non‐native mollusks had a significant effect on *S. obovata* seedling survival with 33% mean difference in seedling density by plot treatment (i.e., plots treated with molluscicide and plots exposed to natural field herbivory intensity). The height cutoff that we used to model the influence of herbivory on seedling survival was set to 2.4 cm because that was the mean height distribution of seedlings at time *t *+* *1.

To assess potential effects of herbivory on vital rates of plants >2.4 cm, we recorded percent mollusk herbivory every 6 months over the course of our study. In total, we observed five tagged plants that were grazed by mollusks, only one of which was >2.4 cm. All of the seedlings that were grazed died within the transition time *t *+* *1. The one grazed plant >2.4 cm survived and had comparable growth to immature plants that were not grazed from time *t* to time *t *+* *1. With low observed mollusk grazing for plant >2.4 cm, we made the assumption that mollusk grazing had little or no effect on vital rates of plants >2.4 cm for our model simulations.

### 2.6 Integral projection model

To evaluate the effect of wet season precipitation and mollusk herbivory on *S. obovata* population dynamics, we developed a continuous size‐dependent integral projection model (IPM; Easterling, Ellner, & Dixon, 2000), which includes wet season precipitation, *a*, and mollusk herbivory, *b*, as covariates:

(1) $$n\left(y,\;t+1\right)=\int_{\Omega }\kappa \left(y,\;x,\;a,\;b\right)n\left(x,\;t\right)dx,$$

where the vector $n\left(y,\;t+1\right)$ represents the number of individuals of size *y* at time (*t *+* *1). The vector $n\left(x,\;t\right)$ summarizes the size distribution of the population at time *t*. The κ kernel is the non‐negative surface of all possible transitions (i.e., survival, growth, and fecundity) of individual plants from size *x* at time *t* to size *y* at time $\left(t+1\right)$, which is composed of two functions, survival‐growth function $p\left(y,\;x,\;a,\;b\right)$ and fertility $f\left(y,\;x,\;a,\;b\right)$.

The survival‐growth function $p\left(y,\;x,\;a,\;b\right)$ represents the probability that individuals of size *x* survives *s*(*x, a*) and grows *g*(*y, x, a, b*) to size *y*:

(2) $$p\left(y,\;x,\;a,\;b\right)=s\left(x,\;a,\;b\right)g\left(y,\;x,\;a,\;b\right).$$

The fertility function $f\left(y,\;x,\;a,\;b\right)$ is calculated using the following equation:

(3) $$f\left(y,\;x,\;a,\;b\right)=s\left(x,\;a,\;b\right)f_{f}\left(x,\;a,\;b\right)f_{n}\left(x,\;a,\;b\right)p_{g}p_{e}\left(x,\;a,\;b\right)f_{d}\left(y\right),$$

where $s\left(x,\;a,\;b\right)$ is the probability of mature plant survival, $f_{f}\left(x,\;a,\;b\right)$ is probability of fruiting, $f_{n}\left(x,\;a,\;b\right)$ is the number of fruits produced, $p_{g}p_{e}\left(a,\;b\right)$ is the probability of germination and seedling establishment, and $f_{d}\left(y\right)$ is the size distribution of seedlings. For our model, we combined $f_{n}\left(x,\;a,\;b\right)$ and $p_{g}p_{e}\left(a,\;b\right)$ to capture reproductive output in terms of number of seedlings produced per mature plant, which is referred to as $p_{r}\left(a,\;b\right)$. The kernel κ is integrated numerically over all possible plant height Ω, using the mid‐point rule (Ellner & Rees, 2006). The result is a large 150 × 150 matrix, which has mathematical properties similar to matrix projection models (see Caswell, 2001). The dominant eigenvalue of this matrix represents the long‐term population growth rate λ, which was calculated using the *popbio* package in R version 3.1.0 (Stubben & Milligan, 2007).

To evaluate the effects of density dependence on plant vital rates, we categorized the tagged plants as either isolated seedlings or seedling clusters. We then tested whether there was a significant difference in the size‐dependent survival and growth for these two categories. We assumed lower survival rate or slower growth of seedlings in clusters than isolated individual seedlings would indicate density dependence. However, we found no significant differences in vital rates of seedlings in clusters and isolated individual seedlings. Therefore, we developed our model without including density dependence.

As interannual precipitation influenced multiple vital rates, we also conducted a life table response experiment (LTRE) analysis to identify the size‐dependent contribution of vital rates to a decrease in population growth rate λ in years with low wet season precipitation, relative to years with high wet season precipitation. The kernel of vital rates contributions $C^{d}$ was calculated (Caswell, 2001; Elderd & Doak, 2006):

(4) $$C^{d}=D^{d}\circ S_{k^{i}},$$

where $D^{d}$ is the difference between the high precipitation and low herbivory kernel $\kappa^{\mathrm{HP}-\mathrm{LH}}$ and the low precipitation and low herbivory kernel $\kappa^{\mathrm{LP}-\mathrm{LH}}$. The $S_{k^{i}}$ kernel represents the sensitivity of the “midway” kernel between $\kappa^{\mathrm{HP}-\mathrm{LH}}$ and $\kappa^{\mathrm{LP}-\mathrm{LH}}.$

### 2.7 Simulations of population dynamics under varying levels of precipitation and herbivory

The IPM model described in Equation (1) was used to project the population dynamics for four scenarios: (1) high wet season precipitation and low herbivory, (2) high wet season precipitation and high herbivory, (3) low wet season precipitation and low herbivory, and (4) low wet season precipitation and high herbivory. Hereafter the four scenarios are abbreviated as HP‐LH, HP‐HH, LP‐LH, and LP‐HH, respectively.

For the high (HP‐LH and HP‐HH) and low (LP‐LH and LP‐HH**)** precipitation‐dependent vital rate models, we set the precipitation parameter *a* in our IPM kernel (Equation (1)) to 10.4 in and 5.99 in, respectively. The high precipitation value was based on total observed wet season precipitation in 2014–2015, and the low precipitation value was based on the 2015–2016 El Nino Southern Oscillation year. To model the effect of mollusk herbivory on population dynamics, we manipulated seedling survival $s\left(x,\;a,\;b\right)$ of the constructed $p\left(y,\;x,\;a,\;b\right)$ function of our IPM kernel (i.e., plants < 2.4 cm). For the high herbivory models (HP‐HH and LP‐HH), we decreased the $s\left(x,a,b\right)$ function of the low herbivory models (HP‐LH and LP‐LH) by 33%, based on the mollusk suppression using Sluggo. The proportional decrease in seedling survival $s\left(x,\;a,\;b\right)$ of the survival‐growth function $p\left(y,\;x,\;a,\;b\right)$ that we used for the high herbivory models (HP‐HH and LP‐HH) was based on the results reported in Kawelo et al. (2012).

## 3 RESULTS

### 3.1 Effect of precipitation and herbivory on vital rates

**Figure 1:**
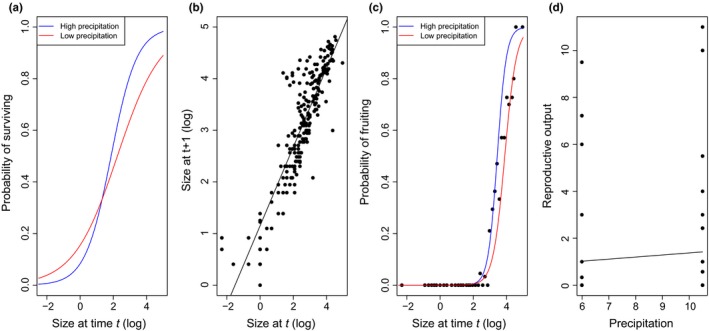
Regression models of vital rates parameters. Panel a = survival *s* (*x, a, b*), b = growth *g* (*x, a, b*), c = probability of fruiting $f_{f}\left(x,\;a,\;b\right)$, d = and reproductive output $p_{r}\left(a,\;b\right)$ (i.e., number of seedling per mature plant). The solid blue line represents high precipitation, and the solid red line represents low precipitation

**Figure 2:**
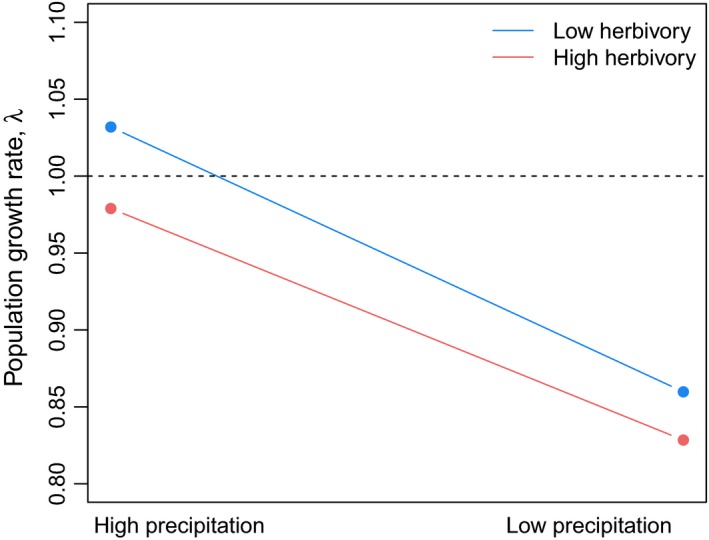
The asymptotic population growth rate λ for scenarios: (1) high wet season precipitation and low herbivory (HP‐LH), (2) high wet season precipitation and high herbivory (HP‐HH), (3) low wet season precipitation and low herbivory (LP‐LH), and (4) low wet season precipitation and high herbivory (LP‐HH)

Mollusk herbivory reduced seedling survival and wet season precipitation and plant size influenced survival, growth, and fertility in contrasting ways (Figure 2). The probability of fruiting increased with precipitation and plant size. Wet season precipitation had a greater effect on the probability of fruiting for larger plants than for smaller plants. The total number of seedlings per mature plant also increased with precipitation but was not influenced by plant size. Two of the plots in 2014 and three of the plots in 2015 contained multiple reproductively mature plants. For these plots, we divide the total number of seedlings by the total number of reproductively mature plants the previous year to calculate $p_{r}\left(a,\;b\right)$, which may have masked the influence of plant size on fertility. We found an interactive effect of plant size and precipitation on survival. Larger plants had higher probability of surviving than smaller plants. Wet season precipitation had mixed effects on survival. With increasing wet season precipitation, the probability of survival increased for large vegetative and reproductive plants but decreased for smaller plants (i.e., seedling; Figure 1).

### 3.2 Population growth rates for varying levels of precipitation and herbivory

**Figure 3:**
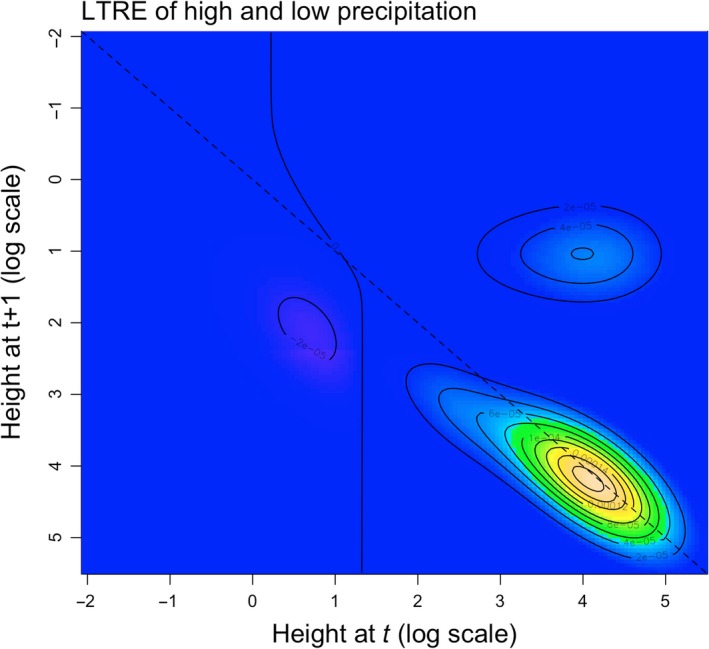
Life table response experiment (LTRE) of *Schiedea obovata,* which decomposes the variation in the population growth rate λ of low wet season precipitation, relative to high wet season precipitation. The dashed line represents survival of *S. obovata*. Area to the left of the solid line represents changes in the life cycle that have a negative effect on λ, and area to the right of the solid line represents changes in the life cycle that have a positive effect on λ

Decreased wet season precipitation had a greater negative effect on *S. obovata* population dynamics than mollusk herbivory (Figure 2). The population growth rates of the four scenarios, HP‐LH, HP‐HH, LP‐LH, and LP‐HH, ranged from 1.032 to 0.828, respectively (Figure 2). The population growth rate of the high precipitation and low herbivory (HP‐LH) scenario was the highest (λ_HP‐LH_ = 1.032), followed by the high precipitation and high herbivory (HP‐HH) scenario (λ_HP‐HH_ = 0.979). The low wet season precipitation scenarios (LP‐LH and LP‐HH) had the lowest population growth rates regardless of herbivory intensity, ranging from λ_LP‐LH_ = 0.86 when herbivory was kept at low intensity to λ_LP‐HH_ = 0.828 when high herbivory was included. Only with high precipitation and low herbivory (HP‐LH) did we find positive population growth for *S. obovata* (λ_HP‐LH_ > 1; Figure 2). We also found that the demographic processes that contributed the most to a decrease in the population growth rate in years with low wet season precipitation was a decrease in the survival of larger vegetative and reproductively mature plants, followed by a decrease fertility and an increase in shrinkage of small vegetative plants (Figure 3).

## 4 DISCUSSION

The effects of environmental stressors on components of plant fitness (e.g., survival and growth) have been well examined (Cowie et al., 2009; Hanley et al., 1995; Joe & Daehler, 2007; Orians et al., 2013; Parmesan, 2006; Pender et al., 2013; Shiels & Drake, 2011). These studies provide insight into the effects of environmental stressors on susceptible vital rates. With this information alone, however, it is impossible to untangle which environmental stressors contribute the most to population decline and species endangerment. For short‐lived organisms, such as *S. obovata*, predictions from ecological synthesis and life history theory suggest that vital rates of earlier life stages will have the greatest impact on population dynamics (e.g., population growth rate) (Silvertown, Franco, Pisanty, & Mendoza, 1993; Stearns, 1992). As a result, it may be expected that environmental stressors that negatively impact earlier life stages will have the greatest negative effect on population dynamics of short‐lived organisms. However, the influence of environmental stressors on population dynamics is dependent on: *which* vital rates are affected, the *magnitude* of change in susceptible vital rates, and the *sensitivity* of those vital rates on population growth rate (Caswell, 2001). Thus, in order to fully understand which environmental stressor, or combination of stressors, drives variation in population growth rate requires a demographic model approach.

In this study, we found that wet season precipitation and initial plant size had varying effects on *S. obovata* vital rates. Wet season precipitation and initial plant size positively influenced survival and the probability of fruiting, with a higher probability of survival and fruiting for large reproductively mature plants than small reproductively mature plants. Consistent with previous studies, we found a positive relationship between survival of later *S. obovata* life stages and interannual precipitation (Dalgleish et al., 2011; Tye et al., 2016). Increased precipitation also had a positive influence on *S. obovata* seedling establishment. The influence of precipitation on the probability of fruiting and reproductive output was also consistent with results from previous studies (Kadmon, 1993; Williams et al., 2015). For earlier life stages (i.e., seedling and small vegetative plants), however, increased wet season precipitation reduced subsequent survival. The underpinning mechanism driving a negative influence of increased wet season precipitation on subsequent survival of early life stages is unknown. However, we offer a plausible explanation for this result. Previous research suggests that seedlings that established in low soil moisture conditions can have a higher root to shoot ratio than seedlings that establish in high soil moisture conditions (Franco, Arreola, Vicente, & Martínez‐Sánchez, 2008; Sánchez‐Blanco, Álvarez, Navarro, & Bañón, 2009). As a result, seedlings that established in low soil moisture conditions can have higher subsequent survival and water use efficiency (Franco et al., 2008; Sánchez‐Blanco et al., 2009). Vegetation flushes following high precipitation events can also intensify competitive interactions, which can lead to decreased seedling survival (Goldstein & Suding, 2014). At our field site, density dependence did not influence seedling survival or population dynamics. However, the role of density dependence may become important for more concentrated populations. Due to the short duration of this study, caution should be taken in interpreting our results to assess restoration outcome. However, as we captured an average wet season precipitation year and a severe El Nino year, with lower than average wet season precipitation, our study provides insight into how declining interannual precipitation over time will likely influence the dynamics of plants across low elevation range distributions.

Based on previous studies of short‐lived species, we predicted that vital rates of earlier life stages (e.g., seedling establishment and fertility) would contribute the most to lower population growth rate in years with low interannual precipitation (Dalgleish et al., 2010). Contrary to our expectation, however, we found that lower survival of larger vegetative and reproductively mature plants, lower fertility, and increased shrinkage of small vegetative plants contributed the most to a lower population growth rate in years with low precipitation (Figure 3). As illustrated by Figure 3, shrinkage of small vegetative plants and lower fertility contributed moderately to lower population growth rate in years with low precipitation, whereas lower survival of larger vegetative and reproductively mature plants had the greatest contribution to lower population growth rate in years with low precipitation. This result illustrates the importance of evaluating the effect of environmental stressors on the full life cycle of an organism in order to fully understand if a targeted environmental stressor is a primary driver of population decline and extinction.

There are a small, but growing, number of studies that have assessed the combined effects of herbivory and abiotic conditions on plant dynamics (Dahlgren & Ehrlén, 2009; Miller et al., 2009; Tye et al., 2016). Variation in insect herbivory pressure constrained the geographical distribution of a long‐lived tree cactus, *Opuntia imbricate* (Miller et al., 2009). Herbivory pressure had varying effects on the population dynamics of the short‐lived herb, *Liatris ohlingerae*, across different habitat types, demonstrating that the effect of herbivory on plant dynamics can be context specific based on local environmental conditions (Tye et al., 2016). Similarly, the effect of harvesting nontimber forest products (i.e., reduction in foliar biomass) on the population dynamics of a long‐lived tree, *Khaya senegalensis*, varied temporally along a precipitation gradient. Regardless of harvest intensity (i.e., high and low), the population growth rate was >1 in the wet region and <1 in the dry region (Gaoue & Ticktin, 2010). Furthermore, abiotic conditions were found to have a greater effect on *K. senegalensis* population dynamics than harvest intensity (Gaoue & Ticktin, 2010). Similar to previous research, we found that alterations in interannual precipitation had a substantially greater effect on plant dynamics than seedling herbivory (Figure 2). We note that the data used to simulate the effect of herbivory pressure on *S. obovata* vital rates and plant dynamics came from a manipulative field experiment that was conducted in close proximity to our study site (Kawelo et al., 2012). Thus, it is reasonable to assume that our models capture an approximant estimate of herbivory pressure at our field site. However, given herbivory pressure was not explicitly quantified both years of the study, it was not possible to assess context‐specific interactions of herbivory across varying abiotic conditions. To evaluate potential synergies between precipitation and mollusk herbivory additional research is needed and should be a focus of future studies.

The results of this research have several applied restoration implications. First, our research illustrates that for short‐lived species like *S. obovata*, a decrease in interannual precipitation can have a greater negative effect on population dynamics than the introduction of non‐native seedling herbivores. Secondly, Kahanahaiki is the driest and lowest range distribution for *S. obovata*. Climate projections suggest wet season precipitation will continue to decrease across *S. obovatas'* geographical range distribution (Giambelluca et al., 2011). As water availability becomes more limited, our results indicate low elevation sites, such as Kahanahaiki, may not be optimal for *S. obovata* restoration. Globally, this research demonstrates how critical it is to quantify the effect of targeted plant–environmental interactions on population dynamics in order to fully understand which environmental factors contribute the most to variation in plant dynamics. Evaluating the strength of plant–environmental interactions on plant dynamics is critical for gaining a mechanistic understanding of what factors drive species decline and developing effective restoration plans.

## CONFLICT OF INTEREST

None declared.

## AUTHOR CONTRIBUTIONS

Both authors contributed to the conception and design of the project. LB collected the field data, constructed the demographic models, and drafted the manuscript. OG contributed significantly to data analysis and manuscript revisions.

## DATA ACCESSIBILITY

Regression coefficients for all vital rates used to construct the IPM models are reported in Table 1.
